# Autoamputation of the Appendix in a Chronic Adnexal Abscess

**DOI:** 10.1155/2018/6010568

**Published:** 2018-02-13

**Authors:** C. Michele Markey, Lauren E. Vestal

**Affiliations:** University of Missouri-Kansas City School of Medicine, Kansas City, MO, USA

## Abstract

Autoamputation of the appendix has rarely been described in the literature. We present a case of a pelvic mass, thought to be a dermoid cyst based on preoperative imaging. After surgical removal and pathological examination, the mass was found to be a chronic pelvic abscess containing the right adnexa as well as an autoamputated vermiform appendix. Differentiating between gynecologic and gastrointestinal disease preoperatively can be difficult and often a definitive diagnosis cannot be made until surgical exploration and pathological review. However, to our knowledge, this is the first described case of a chronic pelvic abscess containing an autoamputated vermiform appendix.

## 1. Introduction

Pelvic masses are frequently seen on imaging. The specific diagnosis of pelvic masses is difficult to determine without surgical removal and pathologic examination. Right lower quadrant masses can be of many different origins, including gynecologic or gastrointestinal origins. Distinguishing between the two can often be difficult [1]. Endometriosis, a common cause of chronic pelvic pain, can involve gynecologic organs, pelvic sidewall, and gastrointestinal organs. We present a case of a pelvic mass that was initially thought to be a right-sided ovarian dermoid tumor, but, after surgical removal and pathologic analysis, was found to be a chronic abscess containing an endometrioma, the right adnexa, and an autoamputated vermiform appendix. Appendiceal involvement of endometriosis is a commonly documented phenomenon. However, autoamputation of the vermiform appendix is rarely reported in the literature. To our knowledge, we present the first case of autoamputation of the vermiform appendix within a chronic abscess also containing an endometrioma, the right fallopian tube, and ovary.

## 2. Case

**Figure 1:**
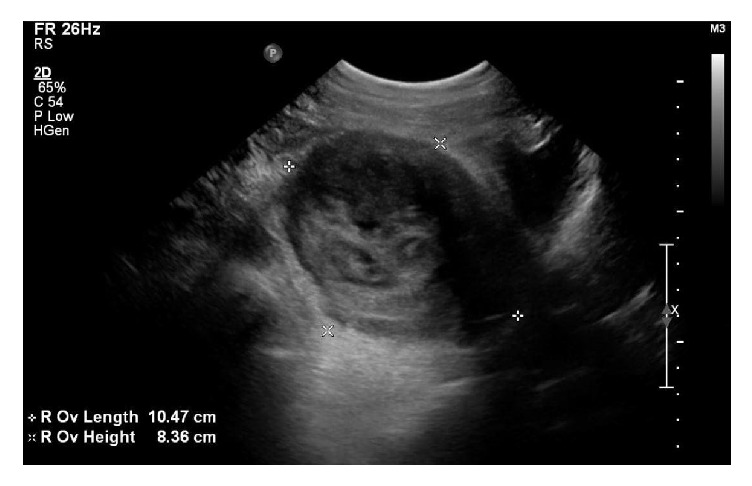
Laparoscopic view of the right adnexal mass with extensive adhesions between the mass and the bowel bilaterally.

A 26-year-old gravida one, para one female without significant past medical history presented to the Emergency Department from a community clinic due to concern for appendicitis. She had been seen at a community clinic earlier that day for chronic right lower quadrant pain of two months' duration. An ultrasound was performed at that community clinic showing a large right adnexal mass. On presentation to the Emergency Department, the patient was afebrile with normal vital signs. Her exam was significant for fullness of the right adnexa and mild tenderness to palpation of the right adnexa. No images were available of the previously obtained ultrasound and repeat imaging was obtained while the patient was in the Emergency Department. Repeat ultrasound showed a heterogeneous right adnexal mass, part solid-part cystic in appearance, measuring 12.6 × 8.2 × 7.9 cm with internal vascularity (Figure 1). Lab work showed anemia; however, white blood cell count was normal. Due to patient's young age and low suspicion for malignancy, epithelial ovarian cancer tumor markers were not obtained. Inhibin A and B levels were assessed and found to be normal. As there was no preoperative concern for infection, C-reactive protein levels and sedimentation rate were not assessed. Based on imaging, the mass was thought to be a dermoid cyst and surgical removal was planned.

**Figure 2:**
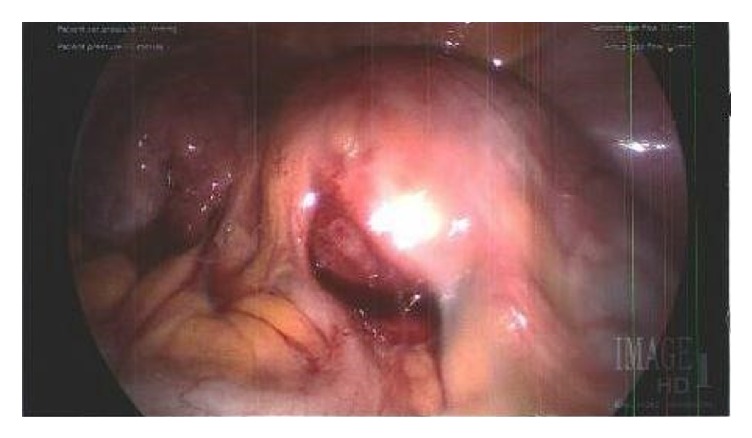
Ultrasound of heterogenous right adnexal mass measuring 12.6 × 8.2 × 7.9 cm, described as part solid-part cystic in nature.

On the day of surgery, the patient denied any changes in her health history and reported continued right lower quadrant abdominopelvic pain. She was taken to the operating room for a planned laparoscopic cystectomy. Upon laparoscopic entry into the abdomen, the pelvic mass was noted to be densely adherent to the uterus, the right pelvic sidewall, and bowel epiploica (Figure 2). The right fallopian tube and ovary were not able to be visualized and were suspected to be encompassed within the mass. The right ureter was also visibly coursing through the mass. Attempt was made to locate the vermiform appendix but was unsuccessful.

Due to dense adhesions that could not be fully addressed laparoscopically, laparotomy was performed. Delineation between the bowel margins and pelvic mass was difficult to distinguish and General Surgery was consulted intraoperatively for assistance. During the lysis of adhesions, rupture of the mass occurred and purulent fluid was noted. The mass was then completely drained and the abscess capsule was removed from the surrounding tissues. No bowel resection was required, as the mass was able to be dissected from the epiploica and did not involve bowel serosa. Attempt was again made to locate the vermiform appendix; however, the appendix was unable to be found.

At the time of abscess rupture, the Anesthesia team reported newly purulent urine present in the Foley catheter. A urinalysis was sent, which later returned without signs of infection. A cystoscopy was performed revealing polypoid inflammatory formations of the internal bladder mucosa adjacent to the site of previous pelvic abscess. No efflux of urine was noted at the right ureteral opening. Urology was consulted for concern for ureteral damage and a right retrograde ureteropyelogram was performed. The retrograde ureteropyelogram showed no evidence of dye extravasation; only mild hydroureter was noted. The case was completed thereafter.

The patient's postoperative course was unremarkable. Piperacillin-tazobactam was administered for 24 hours after surgery due to intraoperative spillage of the abscess. She was then started on an oral amoxicillin/clavulanic acid course. She was discharged home on postoperative day two with the presumed diagnosis of chronic tuboovarian abscess.

She was then seen in clinic two weeks later for follow-up after completing her antibiotic course. At the follow-up visit, she was doing well and reported resolution of her pain. Pathology was reviewed and was consistent with an endometrioma with fallopian tube and ovarian tissue present. Intestinal gland-lined mucosa was identified and determined to be an autoamputated vermiform appendix. Adhesions, bacteria, and acute and chronic inflammation were also present.

## 3. Discussion

Although appendiceal endometriosis is reported many times within literature, reports of autoamputation of the appendix are rare. A case of autoamputation of a mucocele of the appendix was described in 1951 [2]. The mucocele was described as occupying the entire appendix, which had self-amputated from the cecum. Autoamputation of the appendix was also found in a two-year-old who presented for bloody stools with a known history of bowel resection secondary to necrotizing enterocolitis. Imaging showed a calcified abdominal mass that was surgically removed and later found to contain appendiceal mucosa on pathologic examination [3]. To our knowledge, our case is the first of an autoamputation of the appendix secondary to a chronic abscess involving an endometrioma, right fallopian tube, and ovary.

As the clinical presentation and imaging findings for tuboovarian abscesses and appendicitis are similar, these diagnoses can often be confused for one another [1]. Niwa and Hiramatsu describe a case of appendiceal diverticulitis resulting in a pelvic pseudocyst. Initial diagnosis was appendiceal perforation and resultant pelvic abscess based on clinical presentation and imaging; however, after surgical and pathological evaluation, the diagnosis was changed to a pelvic pseudocyst secondary to appendiceal diverticulitis [4]. Interestingly, in a small study of extraovarian condition which mimicked ovarian cancer, 17.7% of these extraovarian conditions were pelvic abscesses [5].

## 4. Conclusion

We present what we believe is the first case to be reported in literature of autoamputation of the vermiform appendix secondary to a chronic abscess involving an endometrioma, as well as the right fallopian tube and ovary. While this particular clinical scenario is rare, it is not uncommon to have difficulty distinguishing between certain gynecologic and gastrointestinal diseases, specifically tuboovarian abscesses and acute appendicitis. This case highlights the potential for intermixing of both the gynecologic and gastrointestinal systems in a chronic pelvic abscess and advocates for involvement of both surgical specialties in these types of cases.
